# Mapping evidence on predictors of adverse sexual and reproductive health outcomes among young women in South Africa: A scoping review

**DOI:** 10.4102/phcfm.v13i1.3091

**Published:** 2021-11-18

**Authors:** Obasanjo A. Bolarinwa, Tlou Boikhutso

**Affiliations:** 1 Discipline of Public Health Medicine, College of Health Sciences, University of KwaZulu-Natal, Durban, South Africa

**Keywords:** predictors, adverse, sexual and reproductive health outcomes, unintended pregnancy, STIs/HIV, South Africa

## Abstract

**Background:**

Globally, most young women have an unmet need for sexual and reproductive health (SRH), which remains a public health concern. Identifying the predictors can help reduce this challenge.

**Aim:**

This scoping review maps evidence on predictors of adverse sexual and reproductive health outcomes among young women in South Africa.

**Method:**

Askey and O’Malley’s framework guided this review. We searched five databases from January 2000 to December 2020 using relevant keywords, Boolean terms and medical subject heading terms. All relevant extracted data were organised into the study themes, and summary of all the findings were reported in a narrative format.

**Results:**

Nine studies met the inclusion criteria out of 1219 studies identified. Four out of the nine studies were national-based studies, while the remaining five studies were conducted in Western Cape (two), Eastern Cape (two) and KwaZulu-Natal (one). Out of the nine studies included, three reported predictors of unintended pregnancy, while six reported predictors of sexually transmitted infections and HIV among young women in South Africa. The most prevailing predictors of adverse sexual and reproductive health outcomes were gender-based violence and alcohol use, while other predictors were lower socio-economic status, place of residence, multiple sexual partnerships, low education and being between the ages of 20–24 years.

**Conclusion:**

We conclude that gender-based violence and alcohol abuse are the most prevailing predictors of adverse sexual and reproductive health outcomes among young women in South Africa.

## Background

Globally, the majority of young women have an unmet need for sexual and reproductive health, which remains a public health concern.^1,2,3^ While barriers in accessing sexual and reproductive health services have been reported as the major risk factor in not meeting young women’s sexual and reproductive health needs,^4,5^ limited access to sexual and reproductive health services and other sexual and reproductive health risk factors such as low knowledge of sexual and reproductive health service and low educational attainment have been linked to adverse sexual and reproductive health outcomes among young women.^6,7,8^

Sexual and reproductive health is a complete state of physical, emotional and social well-being of the sexual and reproductive system.^9^ This means that people can have a satisfying and safe sex life, the capability to reproduce and the freedom to decide if, when, and how often to do so.^9^

There is evidence that when unintended pregnancy and sexually transmitted infection/human immunodeficiency virus (STI/HIV) are given the utmost attention, it can improve young women’s sexual and reproductive health globally, including sub-Saharan Africa (SSA).^10,11,12^ An increase in unsafe abortion,^13^ continuous school dropout^14^ and adverse pregnancy outcomes^15^ are more likely to be more pronounced among young women in SSA as a result of unintended pregnancy.^16^ In the same vein, non-utilisation of condoms during most recent sexual intercourse and multiple sexual partners has been reported as major risk factors driving the high prevalence of STIs/HIV in SSA.^17,18,19^

A study conducted in SSA estimated a 29% of unintended pregnancies among women of reproductive age between 15 years and 49 years, while young women between 15 and 24 years contributed 32%.^20^ In 2020, an estimated 417 million young people had herpes simplex virus type 2 (HSV-2) infections.^21^ World Health Organization (WHO) estimated an annual 357 million new episodes of four curable STIs, including chlamydia, gonorrhoea, syphilis and trichomoniasis globally.^22,23^ Globally, 15% of women living with HIV are between the age of 15–24 years, of whom 80% reside in SSA, and for more than 30 years, this age group has remained much at higher risk of HIV infections than their male counterparts, thus reporting more than 380 000 new HIV infections yearly.^24^

South Africa has been identified as one of the countries in SSA contributing significantly to the continuous increase in adverse sexual and reproductive health outcomes in terms of unintended pregnancy and STIs/HIV among young women.^25,26,27^ This might be because of the high prevalence of early sexual debut among this cohort,^28^ with about 41% of unintended pregnancies reported in a recent study.^27^ Sexually transmitted infections and HIV prevalence have also been higher among South African young women, with approximately 9.8% STIs prevalence^29^ and 21.0% HIV prevalence.^29^

Furthermore, some studies argued that young women’s socio-demographic, economic and structural characteristics in South Africa are some of the risk factors influencing adverse sexual and reproductive health outcomes.^30,31^ However, no study in South Africa has synthesised evidence from the primary studies available on adverse sexual and reproductive health outcomes with a focus on unintended pregnancy and STIs/HIV using scoping review, which is important in actualising sustainable development goal 3 (SDG 3) by the year 2030,^32^ global agenda goal of 90-90-90 towards the eradication of HIV^33^ and other national strategic plans.^34,35^

Thus, this scoping review maps evidence on predictors of adverse sexual and reproductive health outcomes among young women in South Africa. The results from this review will give an account of the prevailing factors predicting adverse sexual and reproductive health outcomes in terms of unintended pregnancy and STIs/HIV among young women in South Africa. Additionally, the result could also shape the direction of the national policymakers in developing targeted interventions that will address the adverse sexual and reproductive health outcomes among young women in South Africa, as no study in South Africa has been able to underscore this.

## Methods

### Design

We adopted the Arksey and O’Malley guidelines methodological frameworks^36^ in designing this study, and we followed the checklist of Preferred Reporting Items for Systematic Reviews and Meta-analyses extension for scoping reviews (PRISMA-ScR) to report this study’s findings.^30,31^

### Research question

This study research question was: ‘What the predictors of adverse sexual and reproductive health outcomes, including unintended pregnancy, STIs/HIV among young women aged 15–24 in South Africa are?’

The eligibility criteria for potential articles to address the research question were determined using the population concept context (PCC) framework, as depicted in Table 1^30^.

**TABLE 1:** The population concept context framework.

Criteria	Determinants
Population	Young women between the age of 15–24 years
Concept	Predictors of adverse sexual and reproductive health outcomes in terms of unintended pregnancy and STIs/HIV.
Context	Studies from 2000 to 2020 in South Africa
Language	English language

Source: Tricco AC, Lillie E, Zarin W, et al. PRISMA extension for scoping reviews (PRISMA-ScR): Checklist and explanation. Ann Intern Med. 2018;169(7):467 -473. https://doi.org/10.7326/M18-0850

STIs/HIV, sexually transmitted infections/human immunodeficiency virus.

### Literature search strategy

We searched for publications on predictors of adverse sexual and reproductive health outcomes in terms of unintended pregnancy and STIs/HIV among young women in South Africa between January 2000 and December 2020. A preliminary search was performed on PubMed using the keywords ‘predictors’ AND ‘sexual and reproductive health’ AND ‘young women’ AND ‘South Africa’ to ensure a comprehensive search. The returned index terms on the search were then used to develop comprehensive terms using the peer review of electronic search strategies 2015 guideline checklist to assess, evaluate and revise our comprehensive vocabulary before performing our main search. We then searched using all identified Medical Subject Headings (MeSH) terms to ensure that all relevant studies were included. The following databases were searched African journals online, web of science, Scopus, PubMed and CINAHL.

To identify grey literature, WHO, United Nations Population Fund (UNFPA) and Guttmacher web pages were searched, while Google Scholar was searched for published theses. Finally, we searched the reference list of all review studies found in the search to determine more eligible studies. Eligible studies were exported to the Endnote version X9’s library for abstract and full article screening after the keyword search and title screening. Articles published between January 2000 and December 2020 were included.

### Eligibility criteria

The following inclusion criteria were applied: (1) studies on unintended pregnancy predictors among young women in South Africa; (2) studies on STI predictors among young women in South Africa; (3) studies on HIV predictors among young women in South Africa; (4) grey literature (conference proceedings, theses, dissertations and government reports) on unintended pregnancy, and STIs/HIV predictors among young women in South Africa; (5) studies conducted among young women and men who reported the studies’ outcomes separately; (6) articles published between 2000 and 2020 and (7) article published in English. Exclusion criteria comprised: (1) studies that do not focus on unintended pregnancy and STIs/HIV predictors among young women in South Africa; (2) grey literature or articles published prior to 2000 and after 2020; (3) studies available in languages other than English; (4) studies with the target population of young women below 15 years and above 24 years and (5) studies with a focus on young men.

### Study selection

After the retrieved articles were exported to EndNote X9’s library, the study selection was in three phases. An independent reviewer conducted the title screening and duplicates removal. Another reviewer conducted the abstract and full article screening. Two independent reviewers were involved in this process by following the study inclusion and exclusion criteria. The research question derived from the eligibility criteria was used to develop a screening form used throughout the screening process. In cases of unresolved disagreement, another member of the research team (T.B.) provided the third opinion through a thorough discussion on why an article should be included or excluded.

### Charting and extracting the data

A data charting form was developed to help determine the suitable variables to be extracted to answer the study research question. Data from included studies were extracted using the following domain author and publication year, study aim, study province or country, study design, research method, sample size (*n*), study population, age group, predictors of unintended pregnancy, predictors of STIs and HIV (key and significant findings)

### Collating and summarising

We employed descriptive statistics to summarise the studies’ key findings, locations and focus using Microsoft Excel 2020, while NVivo version 12 was used to perform content analysis.

### Quality of evidence

A mixed-method assessment tool (MMAT), version 2018, was adopted and piloted by the two independent reviewers to assess the consistency of the selected studies.^33^ The MMAT was used to determine the study’s aim, adequacy, methodology, study design, data collection, study selection, data analysis, results presentation, author discussions and conclusions. An overall quality percentage score for each of the included studies was calculated, and scores were interpreted as high quality (76% – 100%), average quality (51% – 75%) and low quality (≤ 50%). Irrespective of the score, all assessed studies were included.

### Ethical considerations

This study was approved by the Humanities and Social Science Research Ethics Committee of the University of KwaZulu-Natal on the 21st of February 2021, with protocol reference number HSSREC/00002192/2020.

## Results

**FIGURE 1:**
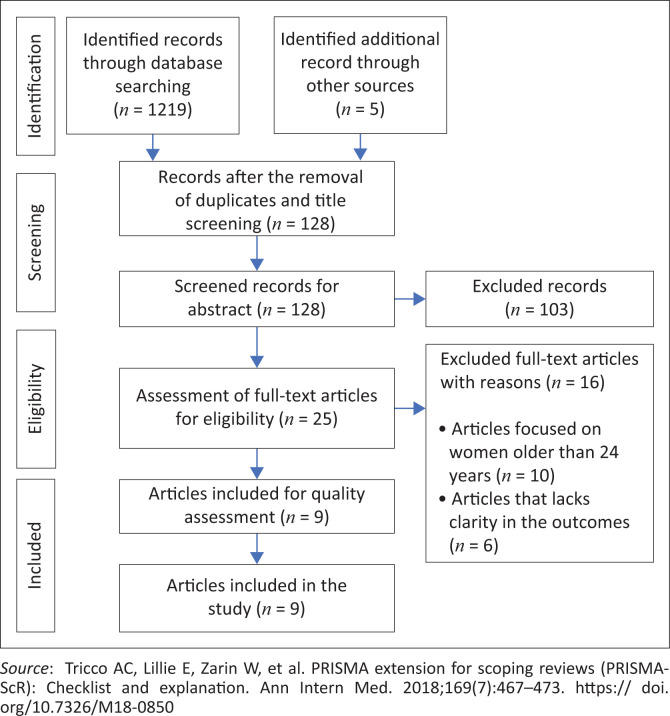
Preferred Reporting Items for Systematic Reviews and Meta-analyses 2009 flow diagram.

The PRISMA flow diagram depicted (Figure 1^36^) shows the process of article screening, inclusion and exclusion. The initial search yielded 1224 results, from which we excluded 1096 because of duplicates and title unconformity. A total of 25 studies were assessed for full-text eligibility, which led to further exclusion of 16 that did not meet our inclusion criteria, leaving us with nine studies to be included in our analysis.

## Characteristics of the included studies

Four (44%) out of nine studies included were national-based studies,^37,38,39,40^ while the remaining five (66%) studies were conducted in different provinces; Western Cape^41,42^ and Eastern Cape^27,43^ had two studies each and one study was conducted in KwaZulu-Natal.^26^ Six (67%) were cross-sectional study design,^26,27,37,38,39,40^, two (22%) studies followed longitudinal design^41,43^ and one (11%) was a randomised control trial.^42^ All the included studies were quantitative. The sample size of the included studies ranged from 88^41^ to 11 904^40^ respondents. The respondents were aged 15–24.

## Themes from included studies

### Predictors of unintended pregnancy among young women in South Africa

Table 2 indicated that three out of nine included studies reported predictors of unintended pregnancies among young women between the age of 15 years and 24 years in South Africa.^27,37,43^ One of these was conducted among young women in Eastern Cape, South Africa, and reported that young women between 17 years and 24 years, young women who have ever had a life experience of sexual abuse and young women who ever drank alcohol were more likely to report unintended pregnancies.^27^

**TABLE 2:** Study’s findings.

Author(s) and year	Study aim	Study province or country	Study design	Research method	Sample size (*n*)	Study population	Age group	Predictors of unintended pregnancy	Predictors of STIs and HIV
Adler and Wallace^41^	To compare rates of HR-HPV persistence between HIV-infected and HIV-uninfected young women.	Western Cape	Longitudinal	Quantitative	88	HIV-infected and HIV-uninfected young women	17–21	-	HR-HPV persistence is higher among young women who were HIV-positive both at 6 and 12 months follow-up. HIV-infected youth were slightly older (mean age 19.91 years, (s.d. = 1.13)
Ajayi and Ezegbe^27^	To estimate the magnitude of unintended pregnancy among AGYW and also examined the effect of sexual violence on unintended pregnancy	Eastern Cape	Cross-sectional	Quantitative	451	Among university young women	17–24	Young women between ages 17–24 years Young women who have had a life experience of sexual abuse Young women who ever drank alcohol	-
Christofides and Jewkes^44^	Aimed to describe the range of risk and protective factors for incident unwanted and unplanned pregnancies occurring over 2 years of follow-up also investigated the relationship between gender inequality and gender-based violence and subsequent unplanned and unwanted pregnancies	Eastern Cape	Longitudinal	Quantitative	136	Cohort of adolescent women	15–18	Physical abuse was a risk factor for unwanted pregnancies Lower socio-economic status Believing that the teenage girl and her boyfriend were mutual main partners	-
Francis and Mthiyane^26^	To investigate the acceptability and feasibility of home-based sampling of STIs and bacterial vaginosis (BV) among young people aged 15–24 years, and to measure prevalence and factors associated with STIs/BV	KwaZulu-Natal	Cross-sectional	Quantitative	248	Young women	15–24	-	Young women in school or working were less likely to have STIs/BV Young women who had ever drunk alcohol were more likely to have STIs/BV Young women who were involved in Genital touching were more likely to have STIs/BV Young women having HSV-2 touching were more likely to have STIs/BV
Ibisomi and Odimegwu^37^	This study examined the distribution of, and factors associated with unintended pregnancy among South African youth.	South Africa	Cross-sectional	Quantitative	1395	Young women	15–24	Young women aged 20–24 years were less likely to report unintended pregnancies Young women with higher education were more likely to report unintended pregnancies Not living together with a spouse were more likely to report unintended pregnancies Residing in the KwaZulu-Natal province were more likely to report unintended pregnancies Having a regular partner were more likely to report unintended pregnancies	-
Mabaso and Sokhela^38^	To investigates socio-demographic and behavioural determinants of HIV infection among AGYW in South Africa.	South Africa	Cross-sectional	Quantitative	3092	Adolescent girls and young women	15–24	-	Young women aged 20–24 years were more likely to report being HIV-positive AGYW who reported condom use at last sexual intercourse were more likely to report being HIV-positive AGYW with Sexual partner within 5 years of age were less likely to report being HIV-positive AGYW with tertiary level education were less likely to report being HIV-positive Low risk of alcohol use was less likely to report being HIV-positive AGYW with one sexual partner were less likely to report being HIV-positive
Menezes and Pokharel^42^	To estimate the prevalence and describe the patterns of concurrent HPV and STIs and associated factors among HIV-negative	Western Cape	Randomised control trial	Quantitative	388	Young women	16–24	-	Young women involving in alcohol use were more likely to report having concurrent HPV/STIs Young women who reported having a sexual partner with STIs were more likely to report having concurrent HPV/STIs
Pettifor and Levandowski^39^	To identify risk factors for HIV infection among young women aged 15–24 years reporting one lifetime partner in South Africa.	South Africa	Cross-sectional	Quantitative	1708	Young women with a one lifetime sexual partner	15–24	-	Young women who had not completed high school were more likely to be infected with HIV Young women between the age of 20–24 were more likely to be infected with HIV
Pettifor and Rees^40^	To determine the prevalence of HIV infection, HIV risk factors and exposure to national HIV prevention programmes, and to identify factors associated with HIV infection	South Africa	Cross-sectional	Quantitative	11 904	Sexually experienced youth	15–24	-	Young women with a history of unusual vaginal discharge in the past 12 months were more likely to report being HIV-positive Young women who were black race were more likely to report being HIV-positive Young women residing in urban areas were more likely to report being HIV-positive Young women between the age of 20 and 24 years were more likely to report being HIV-positive Young women with education below high school level were more likely to report being HIV-positive
Pettifor and Rees^40^	To determine the prevalence of HIV infection, HIV risk factors and exposure to national HIV prevention programmes, and to identify factors associated with HIV infection	South Africa	Cross-sectional	Quantitative	11 904	Sexually experienced youth	15–24	-	Young women who have been sexually active for more than 12 months were more likely to report being HIV-positive Young women with additional Sexual life partners were more likely to report being HIV-positive Young women who were involved in condomless sex at the most recent sex were more likely to report being HIV-positive

HR-HPV, high risk-human papilloma virus; HIV, human immunodeficiency virus; STIs, sexually transmitted infections; AGYW, adolescent girls and young women; s.d., standard deviation; HPV, human papilloma virus.

The second was a longitudinal cohort study conducted among adolescent girls aged 15–18 years. The study showed that adolescent girls who had lifetime physical abuse, those within the lower socio-economic status and the assertion that teenage girls and her boyfriend were main mutual partners were predictors of unintended pregnancies.^43^

The other was a nationwide representative cross-sectional study conducted among young women between the age of 15 years and 24 years in South Africa. The study concluded that young women with higher education, those not living together with a spouse, young women residing in the KwaZulu-Natal province and those having regular partners were more likely to report unintended pregnancies.^37^

### Predictors of STIs/HIV among young women in South Africa

Six studies reported predictors of HIV and AIDS among young women in South Africa,^26,38,39,40,41,42^ as shown in Table 2. A longitudinal study conducted in the Western Cape among HIV-positive and negative young women between the age of 17–21 years reported that persistence of high risk-human papilloma virus (HR-HPV) occurred higher among young women who were HIV-positive both at 6 and 12 months follow-up compared to HIV-negative young women.^41^

One was a cross-sectional study conducted among young women aged 15–24 years in KwaZulu-Natal, South Africa, with the aim of investigating the acceptability and feasibility of home-based sampling of STIs and Bacterial Vaginosis (BV) and to measure prevalence and factors associated with STIs/BV. The study concluded that young women in school or those working were less likely to experience STIs/BV. However, young women who had ever drunk alcohol, those involved in genital touching and young women with HSV-2 were more likely to have STIs/BV.^26^ The randomised control trial of 388 young women between the ages of 16 years and 24 years conducted in the Western Cape showed that young women involved in alcohol use and those who reported having a sexual partner with STIs were more likely to report having concurrent HPV/STIs.^42^

The national cross-sectional survey of 3092 adolescent girls and young women (AGYW) in South Africa reported that young women between 20 years and 24 years and AGYW who reported condom use at last sexual intercourse were more likely to report HIV infection. Adolescent girls and young women with sexual partners within five years of age, with tertiary level education, having low risk of alcohol use and having one sexual partner were less likely to report HIV infection.^38^

Another was a wide range of national studies conducted by Pettifor and Rees.^40^ It concluded that young women with higher odds of reporting HIV infection had a history of unusual vaginal discharge in the past 12 months, were of the black race, resided in the urban area and were aged 20–24 years. Besides, they were young women with educational levels below high school, young women who have been sexually active for more than 12 months, with an additional sexual life partner, and young women involved in condomless sex at most recent sex. Similarly, another nationally representative study of HIV infection among 1708 young women aged 15–24 years reporting one lifetime partner concluded that young women who had not completed high school and young women between the age of 20 years and 24 years were more likely to be infected with HIV.^39^

## Discussion

Our scoping review was conducted to map existing literature on the predictors of adverse sexual and reproductive health outcomes among young women in South Africa.

Three (33%) of the nine included studies reported predictors of unintended pregnancy among young women in South Africa.^27,37,43^ Sexual abuse, use of alcohol,^27^ physical abuse,^37,43^ an assertion on the main mutual partnership, lower socio-economic status,^43^ higher educational attainment, living arrangement, provincial residence and having a regular partner^37^ were the identified predictors.

Physical abuse as a predictor of unintended pregnancy was reported twice in the study by Christofides and Jewkes^43^ and Ibisomi and Odimegwu,^37^ signifying a key predictor of unintended pregnancy among young women in South Africa. This is similar to a study conducted among Bangladeshi women who concluded that unintended pregnancy is higher among women who had ever experienced physical violence,^44^ and the same conclusion was reported in a similar study conducted in Peru among pregnant women.^45^

Sexual abuse and alcohol use were identified as predictors of unintended pregnancy among young women in South Africa.^27^ This is in concordance with a study conducted in Nepal by Acharya and Paudel^46^ who concluded that young women who had ever experienced sexual violence had a higher likelihood (2.3%) of reporting unintended pregnancy. Similarly, a study conducted by Ahinkorah and Seidu^47^ in 22 SSA countries also linked sexual violence to unintended pregnancy. In the same vein, a self-reported periconceptional substance use study conducted among women reported that women who use alcohol were more likely to report unintended pregnancy.^48^

We found that higher educational attainment, not living together with a spouse, residing in the KwaZulu-Natal province and having a regular partner were predictors of unintended pregnancy among young women in South Africa.^37^ These predictors are similar to the conclusion of a study conducted on individual and contextual factors associated with unintended pregnancy among 6791 AGYW (aged 15–24 years) in 10 SSA countries.

We found higher education attainment and variation in type place of residence as predictors of unintended pregnancy among young women in South Africa.^49^ Women’s level of education has always been a critical factor in decision-making, and the impact of educational attainment on sexual and reproductive health, particularly unintended pregnancy, has been discussed in the past.^50^ This study result aligns with a study conducted in Ghana by Ameyaw^51^ and a study conducted in Ethiopia by Habte and Teklu.^52^ As young women with a higher level of education are often employed, it could be that the young women often miss their family planning service appointment or find it difficult to plan their reproductive health needs because of work pressure.^53^

Lower socio-economic status was also a predictor of unintended pregnancy identified in this study.^43^ This finding is in line with a study conducted by Iseyemi, Zhao^54^ that women with lower socio-economic status tend to report unintended pregnancy compared to their counterparts with higher socio-economic status. This finding might be because young women with lower socio-economic status could not make independent financial decisions or could not afford suitable contraception methods.^49,55^

The remaining six studies included in this study reported predictors of STIs/HIV among young women in South Africa.^26,38,39,40,41,42^ The study findings show that being HIV-positive was reported as a predictor to HR-HPV among young women in South Africa.^41^ Alcohol use, touching of the genital area, being HSV-2 positive were predictors of having STIs/BV^26^ and having a sexual partner with STIs was reported as a predictor of having concurrent HPV/STIs.^42^ Similarly, a study conducted by Seth and Wingood^56^ among young African American women also identified alcohol use as a strong predictor of having STIs. In the same vein, being exposed to any form of STIs, including HIV and HS-2, has been reported to be a predictor of having any other form of STIs in the later life of young women.^57^

Furthermore, our study reported that the use of condoms during sexual intercourse in the last 5-year was reported as a predictor of HIV,^38^ young South African women whose population group were black, those who have been reporting unusual vaginal discharge in the past 12 months, those residing in the urban area, those with lower educational attainment, being sexually active, having multiple sexual partners, those involved in condomless sexual intercourse^40^ and young women between the age of 20–24 years were more likely to report having HIV.^39^

This study’s results are in corroboration with a study conducted in Uganda by Nankinga and Misinde,^58^ which concluded that having multiple sexual partners remains one of the strong predictors of having STIs, including HIV. Similarly, another study conducted in Ethiopia supported these findings.^59^ We also identified involving in condomless sexual intercourse as a predictor of reporting being HIV-positive among young women in South Africa; however, a study conducted in Malawi opposed this finding as the study reported that there is no significant relationship between condom use during sex and being HIV-positive,^60^ while another study conducted in Uganda supported our findings that condom use during sexual intercourse reduces the likelihood of being HIV-positive.^61^ However, our study also reported that condom use during sexual intercourse in the last 5-year could be a risk factor for HIV.^38^ This may be that the practice of condom use started after the young women had contracted the virus as such condomising during sexual activities could halt the transmission of the virus to their sexual partners.^62^

Finally, our findings indicated that young women in South Africa between the age of 20–24 years, those with less education and those residing in urban areas were more likely to report being infected with HIV. These findings are in line with mapping studies conducted in Uganda and Malawi by Chimoyi and Musenge^61^ and Nutor and Duah,^63^ respectively, concluded that young women who reside in the urban area, those less educated and those between the age of 20–24 years have higher odds of reporting being HIV-positive compared to their counterparts who reside in a rural area, more educated and those between the of age 15–19 years.

## Implication for policy and public health

This study’s findings are relevant to policy and public health. Predictors of adverse sexual and reproductive health outcomes among young women in South Africa identified in this study are important in developing required interventions and policies to halt its continuous increase. Among identified predictors of adverse sexual and reproductive health outcomes among young women in South Africa, gender-based violence was most prevailing to unintended pregnancy, while alcohol use was identified as the common predictor to both unintended pregnancy and STIs/HIV, and this calls for the attention of policymakers, government agencies, non-governmental organisations and key stakeholders in policy formation and implementation to develop and implement behaviour and social policy that will be targeted at eliminating gender-based violence exposure and alcohol use abuse among young women in South Africa to reduce or eliminate adverse sexual and reproductive health outcomes.

Regarding public health implications, this study’s findings also identified residences (area of residence or province of residence) and other health behavioural factors as predictors of adverse sexual and reproductive health outcomes. This will provide public health providers with the blueprint that will guide their programmes and interventions. Furthermore, it will help ensure optimal utilisation of scarce resources to reduce adverse sexual and reproductive health outcomes, including unintended pregnancy and STIs/HIV among young women in South Africa.

## Implication for research

These research findings identified gender-based violence exposure and alcohol use as the most prevailing predictors of adverse sexual and reproductive health outcomes among young women in South Africa. However, what is unknown is the degree to which these two predictors influence these adverse outcomes at household and community levels; as such future studies should consider conducting primary research that will help the policymakers and stakeholders in understanding the extent to which adverse sexual and reproductive health outcomes among young women in South Africa are being influenced by these predictors (gender-based violence exposure and alcohol use) at both household and community levels.

## Strengths and limitations

This study has numerous strengths. Firstly, scoping review allows different study designs to be included in a study, and this allowed the authors to systematically search and select relevant literature to describe and map the available evidence on adverse sexual and reproductive health outcomes among young women in South Africa. This study design also allowed us to further identify literature gaps useful to inform future research and policy implications. To the best of our knowledge, this study is the first scoping review focusing on adverse sexual and reproductive health outcomes among young women in South Africa in terms of unintended pregnancy and STIs/HIV covering 20 years range. However, there is no study without limitation. The study possibly failed to capture some other relevant articles because we searched fewer databases in the study. Also, we were not involved in the study design and data collection of any of the studies included in this study. Despite these limitations, this review provided evidence useful to guide future research in eliminating or reducing adverse sexual and reproductive health outcomes among young women in South Africa.

## Conclusion and recommendation

This study has revealed evidence of existing literature on adverse sexual and reproductive health outcomes among young women in South Africa while identifying gaps for future research. We conclude that predictors of adverse sexual and reproductive health outcomes (i.e. unintended pregnancy and STIs/HIV among young women in South Africa) were lower socio-economic status, place of residence, multiple sexual partners, lower educational level and being between the ages of 20–24 years. However, the most prevailing predictors among all the identified predictors were gender-based violence (sexual and physical abuse) and alcohol use. This study findings call for the attention of key stakeholders towards policy formation and development of behaviour and social intervention, such as encouraging the young women to speak up after the incidents and constant community engagement by government or non-governmental organisations on the adverse impact of gender-based violence and alcohol use on sexual and reproductive health outcomes of young women in South Africa. The community engagement can be in the form of playlets and one-on-one discussions, while the victims who speak up should be given rewards to encourage other young women to speak up. Particular interest should be channelled towards the marginalised communities in the urban areas and hard-reach areas in rural South Africa.
